# The long-term effect of elexacaftor/tezacaftor/ivacaftor on cardiorespiratory fitness in adolescent patients with cystic fibrosis: a pilot observational study

**DOI:** 10.1186/s12890-024-03069-8

**Published:** 2024-05-28

**Authors:** Nela Stastna, Lenka Hrabovska, Pavel Homolka, Lukas Homola, Michal Svoboda, Kristian Brat, Libor Fila

**Affiliations:** 1 Department of Pulmonology, Second Faculty of Medicine, Charles University and Motol University Hospital, Prague, Czech Republic; 2 Faculty of Medicine, Masaryk University, Brno, Czech Republic; 3 Department of Sports Medicine and Rehabilitation, St. Anne’s University Hospital and Faculty of Medicine, Masaryk University, Brno, Czech Republic; 4 Department of Paediatric Infectious Diseases, University Hospital Brno and Faculty of Medicine, Masaryk University, Brno, Czech Republic; 5 Institute of Biostatistics and Analyses Ltd. and Faculty of Medicine, Masaryk University, Brno, Czech Republic; 6 Department of Pulmonology, University Hospital Brno and Faculty of Medicine, Masaryk University, Brno, Czech Republic

**Keywords:** Cystic fibrosis, Cardiopulmonary exercise testing, Elexacaftor/tezacaftor/ivacaftor

## Abstract

**Background:**

Physical activity is a crucial demand on cystic fibrosis treatment management. The highest value of oxygen uptake (VO_2peak_) is an appropriate tool to evaluate the physical activity in these patients. However, there are several other valuable CPET parameters describing exercise tolerance (W_peak_, VO_2VT1_, VO_2VT2,_ VO_2_/HR_peak_, etc.), and helping to better understand the effect of specific treatment (V_E_, V_T_, V_D_/V_T_ etc.). Limited data showed ambiguous results of this improvement after CFTR modulator treatment. Elexacaftor/tezacaftor/ivacaftor medication improves pulmonary function and quality of life, whereas its effect on CPET has yet to be sufficiently demonstrated.

**Methods:**

We performed a single group prospective observational study of 10 adolescent patients with cystic fibrosis who completed two CPET measurements between January 2019 and February 2023. During this period, elexacaftor/tezacaftor/ivacaftor treatment was initiated in all of them. The first CPET at the baseline was followed by controlled CPET at least one year after medication commencement. We focused on interpreting the data on their influence by the novel therapy. We hypothesized improvements in cardiorespiratory fitness following treatment. We applied the Wilcoxon signed-rank test. The data were adjusted for age at the time of CPET to eliminate bias of aging in adolescent patients.

**Results:**

We observed significant improvement in peak workload, VO_2 peak_, VO_2VT1_, VO_2VT2_, V_E_/VCO_2_ slope, V_E_, V_T_, RQ, VO_2_/HR peak and RR peak. The mean change in VO_2 _peak was 5.7 mL/kg/min, or 15.9% of the reference value (SD ± 16.6; *p*= 0.014). VO_2VT1_ improved by 15% of the reference value (SD ± 0.1; *p*= 0.014), VO_2VT2 _improved by 0.5 (SD ± 0.4; *p*= 0.01). There were no differences in other parameters.

**Conclusion:**

Exercise tolerance improved after elexacaftor/tezacaftor/ivacaftor treatment initiation. We suggest that the CFTR modulator alone is not enough for recovering physical decondition, but should be supplemented with physical activity and respiratory physiotherapy. Further studies are needed to examine the effect of CFTR modulators and physical therapy on cardiopulmonary exercise tolerance.

## Background

Cystic fibrosis (CF) is the genetic disorder affecting the lungs worldwide. There is substantial heterogeneity of clinical manifestation in patients with CF. Cystic fibrosis transmembrane conductance regulator (CFTR) mutation results in the development of bronchiectasis, recurrent infectious exacerbations and lung function decline. Thus, patients moreover have increased dead space (V_D_), which is why the alveolar ventilation is lower compared to healthy subjects. During exercise in CF patients, tidal volume (V_T_) increases inadequately, the respiratory rate (RR) then increases to heighten minute ventilation (V_E_) and reach an adequate oxygen uptake (VO_2_). Response to physical activity is inefficient [1, 2]. Muscle function and muscle mass, the same as cardiac abnormalities, are mostly limitations in mild or moderate CF, patients with severe lung disease, oxygen delivery and non-physiological respiratory mechanics limit exercise capacity [1, 3–7].

Cardiopulmonary exercise testing (CPET) measures aerobic exercise capacity and provides a comprehensive assessment of respiratory, cardiac and musculoskeletal function during exercise and recovery and may be used for prognosis and risk assessment [8]. Not just VO_2_ peak is a predictor of survival, but also peak workload (W peak), V_E_/VO_2_ peak (ventilatory equivalent for oxygen, EQO_2_) and V_E_/VCO_2_ peak (ventilatory equivalent for carbon dioxide, EQCO_2_) may be significant [8].

Ivacaftor and lumacaftor/ivacaftor combination had no effect on VO_2_ peak, only sets of case reports in tezacaftor/ivacaftor demonstrated minor improvements [9–13]. The novel triple combination of the modulators of CFTR channel, elexacaftor/tezacaftor/ivacaftor (ETI) provides substantial clinical improvement and prolonged median predicted survival, improves lung functions (ppFEV_1_ increases by 14.3%); also promotes over-nutrition and overweight, which might affect the physical activity attitude otherwise [14, 15]. The exact quantitative evaluation is still not well known because there is not enough documented evidence of CPET-derived measures of triple combination. The data published so far are not sufficiently convincing. ETI seem to improve VO_2_ peak, but still lack more detailed data about the physical tolerability mechanism [16]. By performing this trial, we try to fill the gap in the knowledge of additional CPET parameters, than just VO_2_ peak. These are important tools to evaluate the effect of the new treatment on prognosis and survival.

We hypothesize that patients with CF will improve their physical activity by several adaption mechanisms, e.g. higher myocardial contractility and cardiac output (HR, higher AT which correlate with decreasing values of RQ and V_E_/VO_2_), more effective gas exchange (increased VO_2_ peak and VCO_2_), lower work of breathing (improved V_E_ due to increased V_T_, RR and decreased V_D_/V_T_), and lower hyperventilation following elexacaftor/tezacaftor/ivacaftor use. Also, the aim of this study is to define other CPET parameters suitable for evaluating the cardiorespiratory demands in cystic fibrosis.

## Methods

### Subjects

This is a prospective observational, non-randomised study. Inclusion criteria comprised a diagnosis of CF based on current guidelines, EMA-approved genotype for ETI indication and informed consent provided by the patient or their legal representative. All the patients were ETI treatment-naïve. For this study, we analysed data of patients with CF aged 8–19 years at the time of the first testing, who had a full CPET meeting between January 23, 2019, and February 9, 2023. The follow-up measurement was performed at least 12 months after ETI commencement (Kaftrio ®, elexacaftor 100 mg, tezacaftor 50 mg, ivacaftor 75 mg; always used in combination with Kalydeko ®, ivacaftor 150 mg). This was the only intervention made; patients haven’t engaged in standardized exercise training.

### Protocol

All the testing was performed during a clinically stable period. We used an exhaustive ramp incremental (10-25W/min) cycling CPET (Ergoline, Ergoselect, Bitz, Germany) protocol. We selected the ramp protocol based on the patient’s physical activity level, body weight and sex. After a 3-min warm-up (10-40W), all the participants completed a test to the point of exhaustion. The protocol was tailored to the individual to yield a fatigue-limited exercise duration of 8–12 min. A five-minute active cool down period followed CPET. Breath-by-breath analysis was provided, and the O_2_ and CO_2_ concentrations of exhaled air with ventilatory volume was measured via face mask with connected gas and flow spirometer sensors. The stress test was performed on the ergometer ERGOLINE, and the exhaled gases was analysed by POWER CUBE – Schiller (Switzerland). A ramp protocol was used while VO_2_, VO_2VT1_, VO_2VT2_, V_E_/VCO_2_, V_E_/VO_2_, V_E_/VCO_2_ slope, V_E_, V_T_, RQ, VO_2_/HR, RR parameters were measured every 10 s, and peak values taken as the highest 15 s achieved during the test. Blood pressure and Sp0_2_ were measured during CPET monitoring.

We evaluated anthropometric parameters: height (cm), weight (kg). Our outcome was to evaluate respiratory CPET-derived parameters: maximal workload (W, W/kg, % ref.), RQ max., VO_2 peak_ (L, mL/kg/min, % ref.,), VO_2VT1_ (L/min, % ref.), VO_2VT2_, (L/min), V_E_/VCO_2_ slope, V_E_ (L/min, % ref.), V_T_ (L, % ref.), V_D_/V_T_ in rest and in maximal effort, RR (min^−1^), VO_2_/HR (ml/beats per minute).

### Statistical methods

Numerical parameters are described by mean (standard deviation = SD). Change during two times is tested by paired Wilcoxon signed-rank test. All tests are two-sided on level of significance 5%. Analysis was prepared in the software R (v4.2) (Bell Laboratories, Inc., Windsor, WI, US).

## Results

Thirteen patients were eligible, but only 10 patients with CF met the criteria for ETI therapy and were included in the final analysis. The mean age was 14 years, mean ppFEV_1_ 89.4%, 70% of the patients with CF were *F508del* homozygotes. Baseline patients’ characteristics are presented in Table 1.

**Table 1:** Baseline characteristics (*N* = 10)

Age, mean (SD) years	14.1 (2.8)
Male, n (%)	8 (80)
Homozygosity *F508del*, n (%)	7 (70)
ppFEV1, mean (SD), %	89.4 (10.9)
Height, mean (SD), cm	164.1 (13.8)
Weight, mean (SD), kg	53.4 (13.2)

*SD* Standard deviation

We observed significant improvements in VO_2 peak_, the mean change was 0.8 L (SD ± 0.6; *p *= 0.002), 15.9% of the reference value (SD ± 16.6; *p* = 0.014). As well as VO_2VT1_ improved by 15% of the reference value (SD ± 0.1; *p* = 0.014) and VO_2VT2_ improved by 0.5 (SD ± 0.4; *p* = 0.01). Patients achieved also better VO_2VT2_, VE/VCO_2_ slope, V_E_, V_T_, RQ, VO_2_/HR_peak_, and RR_peak_ values. Even V_D_/V_T_ marker improved. Only RQ remained unchanged.

Complete data are presented in Table 2. The parameters were recalculated based on age and current weight (% ref.), so the impact of aging was eliminated.

**Table 2:** Mean change between baseline and follow up CPET (*N* = 10)

			**Mean change CPET**	***P*-value**
	Baseline, *N* = 10	Follow up, *N* = 10		
Workload (W) Mean (SD)	178.4 (64.8) (128.0 – 238.2)	202.6 (54.1) (152.5 – 244.0)	24.2 (36.1) (-5.5 – 37.5)	0.074
Workload (W/kg) Mean (SD)	3.2 (0.6) (2.8 – 3.7)	3.1 (0.5) (2.6 – 3.5)	-0.1 (0.4) (-0.3 – 0.0)	0.360
Workload (% ref.) Mean (SD)	96.3 (23.9) (76.2 – 114.2)	99.3 (17.5) (91.5 – 112.8)	3.0 (12.5) (-4.8 – 8.5)	0.575
RQ max Mean (SD)	1.2 (0.1) (1.1 – 1.2)	1.2 (0.1) (1.2 – 1.3)	0.0 (0.1) (0.0 – 0.1)	0.359
VO_2 _peak (L) Mean (SD)	1.9 (0.8) (1.3 – 2.6)	2.7 (0.7) (2.1 – 3.1)	0.8 (0.6) (0.3 – 1.1)	0.002
VO_2 _peak (% ref.) Mean (SD)	78.5 (18.5) (65.2 – 92.0)	94.4 (12.0) (88.2 – 105.0)	15.9 (16.6) (8.2 – 24.2)	0.014
VO_2 _peak (mL/kg/min) Mean (SD)	34.8 (7.7) (29.7 – 41.6)	40.5 (5.7) (35.5 – 45.0)	5.7 (8.1) (0.8 – 9.6)	0.064
VT_1_ (L/min) Mean (SD)	0.7 (0.4) (0.5 – 1.1)	1.0 (0.3) (0.8 – 1.3)	0.3 (0.2) (0.1 – 0.5)	0.019
VO_2V__T1_ (L/min) (% ref.) Mean (SD)	45.9 (0.2) (35.5 – 64.8)	60.9 (0.1) (48.8 – 72)	15 (0.1) (0.6 – 2.4)	0.014
VO_2__V__T__2_ (L/min) Mean (SD)	1.4 (0.6) (0.9 – 2.0)	1.9 (0.6) (1.4 – 2.4)	0.5 (0.4) (0.2 – 0.9)	0.010
V_E_/VCO_2_ slope Mean (SD)	26.2 (4.6) (23.2 – 29.2)	25.5 (5.2) (21.4 – 29.2)	-0.8 (3.6) (-3.1 – 0.5)	0.721
V_E_ (L/min) Mean (SD)	72.1 (34.5) (37.1 – 99.4)	101.7 (29.5) (75.9 – 115.6)	29.6 (28.0) (4.6 – 43.3)	0.027
V_E_ (L/min) (% ref.) Mean (SD)	91.2 (29.5) (68.8.—115)	112.3 (22.4) (102.5—115)	21.1 (29.8) (0 – 40.5)	0.064
V_T_ (L) Mean (SD)	1.6 (0.8) (1.0 – 2.0)	2.1 (0.6) (1.6 – 2.5)	0.5 (0.5) (0.2 – 0.8)	0.006
V_T_ (L) (% ref.) Mean (SD)	60.4 (25.8) (39.2—73)	78.3 (17.1) (72 – 82)	17.9 (13.4) (10.2—25)	0.009
V_D_/V_T_ (rest) Mean (SD)	0.3 (0.1) (0.3—0.3)	0.2 (0.1) (0.2—0.3)	0 (0) (-0.1—0)	0.028
V_D_/V_T_(max.) Mean (SD)	0.2 (0) (0.2—0.3)	0.2 (0) (0.2—0.2)	0 (0) (-0.1—0)	0.038
RR peak (min^-1^) Mean (SD)	46.6 (8.3) (42.0 – 49.2)	48.1 (4.5) (47.0 – 50.0)	1.5 (8.1) (-1.8 – 8.0)	0.552
VO_2_/HR peak (ml/beat) Mean (SD)	10.2 (4.0) (6.9 – 13.1)	14.0 (3.1) (10.9 – 16.7)	3.8 (3.1) (1.6 – 5.1)	0.008

The difference between two examinations is tested using the paired Wilcoxon test

During controlled CPET, all the patients indicated the fatigue of lower extremities as their reason for stopping. We did not observe any exercise-induced arrythmia.

## Discussion

In this study, we report improvement in most of the parameters, which are valuable predictors of death or lung transplant in CF (VO_2 _peak, max. effort, peak work rate, V_E_/VCO_2 _slope) and parameters valuable to understand the ventilatory efficiency (VO_2VT1_, VO_2VT2_, V_E_, V_T_, V_D_/V_T_VO_2_/HR peak and RR peak) [8]. An abnormally low exercise capacity and deconditioning in CF results in VO_2 _peak < 82% predicted and/or peak workload < 93% predicted, VO_2VT1 _occurring < 50% predicted VO_2 _peak [17]. Patients in this cohort achieved improvement in two of these parameters, beyond deconditioning. This might suggest improved prognosis in patients with CF treated with ETI for at least one year.

These data provided on triple combination of CFTR modulators therapy are higher than those achieved on double combination (lumacaftor/ivacaftor or tezacaftor/ivacaftor) in Danish patients with CF followed for the same period of use (VO_2_peak 1.07 mL/min/kg, maximal workload change 14.2W) [18]. Therefore, ETI might be more effective in improving CPET-derived parameters, but improvement is likely multifactorial, and further investigation in a larger patient cohort is necessary.

Older patients with CF aged > 40 years deal with specific comorbidities, while younger patients with CF are healthier than ever due to the variant treatment strategies. Still, physical fitness in CF takes an indisputable position to effect quality of life and prognosis. Medication which reduces the amount of mucus in the respiratory tract and improve pulmonary function could not be the only reason for improved exercise tolerance. Patients eligible into our study were mostly teenagers; the maximal VO_2_ peak increased significantly in absolute but also in relative values, leaving minimal doubts about the role of ageing bias over the study period [17]. Other CPET variables, VO_2VT1_ and VO_2VT2_, demonstrate improved aerobic capacity, physical fitness, and more effective training to the maximal effort. Improvement of V_D_/V_T_ suggests more effective ventilation and decreased V/Q mismatch, as well as improved lung function in general. This would also hint at statistically significant improvement in VO_2_/HR.

In this cohort, the mean ppFEV_1_ is 89.4%; research suggests that in mild lung disease, the respiratory limitation of exercise capacity is rather low [13]. Pulmonary function improvement alone in CFTR modulator users would then be unlikely to change the exercise tolerance. The impact of ETI on the exercise tolerance improvement is hypothesized by several mechanisms [19]. CFTR protein is expressed in myocardial cells, vascular smooth muscle cells, and sarcolemma and sarcoplasm of skeletal muscle cells [20–22]. Impaired CFTR function results in local vasoconstriction and affects nitric oxide production [23].Therefore, the CFTR modulators might improve reduced peripheral O_2_extraction during exercise and utilisation of O_2_not only by skeletal muscle, but it is also suggested, by reduced V_E_/ VO_2 _peak [16]. CFTR protein is hypothesized to be involved in regulating mitochondrial oxidative stress and mitochondrial function in adenosine triphosphate production [24,25].

Decreased systemic inflammation by reducing several interleukins and pro-inflammatory mediators after CFTR modulator use affects cardiorespiratory fitness. Inter alia, chronic inflammation (especially *Pseudomonas aeruginosa* airway infection) relates to impaired aerobic capacity [26, 27]. Systemic inflammation is demonstrated to lead to muscle atrophy and impaired contractility [28].

To examine body composition change, it is necessary to distinguish the mechanism of VO_2_ peak change. The pattern of weight gain (whether muscle or fat) due to triple therapy must be studied. This is because increased adiposity has been suggested to contribute to the decreased VO_2_outcome [19]. Even in this cohort, the patient who gained the most weight (+ 20.4 kg), where BMI increased from 21.86 kg/m^2^ to 28.38 kg/m^2^, reported the highest VO_2 _peak decrease (41.9 mL/kg/min to 35.2 mL/kg/min). Last but not least, it is necessary to consider the change in the mental state of patients on triple therapy and the awareness of new life horizons and possibilities, along with the awareness of the need for more intensive care for overall fitness.

There are several limitations of this study. First, the baseline testing was performed during the COVID-19 pandemic era, so we were not able to recruit more patients in this trial. Even a change in the patient’s physical activity manners during the pandemic era changed to a more sedentary style, which is difficult to quantify. Second, we did not perform a capillary blood gas analysis, and we were therefore not able to assess the V/Q mismatch. Third, the increase in absolute values of certain parameters (e.g. VO_2 _peak) might partly be attributed to the given adolescent’s growth. However, there were significant improvements also in relative values (% of predicted), therefore aging bias (if any) appears to be limited. Comparative trials with a cohort of same-aged CF patients ineligible for ETI would reveal other perspectives, and the risk of a worsened overall status due to severe CFTR pathogenic variants could bias the results.

## Conclusions

We demonstrated improvements in cardiorespiratory fitness in adolescent patients with cystic fibrosis following at least one year of ETI therapy by performing controlled CPET testing. CFTR modulator treatment alone might not be effective in transforming all the mechanisms of exercise intolerance. Understanding the impact of new therapeutical strategies in cystic fibrosis is important for better therapeutical evaluation and survival assessment. Further comparative trials with a larger cohort need to be performed to streamline our results.

## Data Availability

The datasets used and/or analysed during the current study available from the corresponding author on reasonable request.

## Abbreviations

AT: Anaerobic threshold
BMI: Body mass index
CFTR: Cystic fibrosis transmembrane conductance regulator
CF: Cystic fibrosis
CO_2_: Carbon dioxide
COVID-19: Coronavirus disease of 2019
CPET: Cardiopulmonary exercise testing
EQO_2_: Ventilatory equivalent for oxygen
EQCO_2_: Ventilatory equivalent for carbon dioxide
ETI: Elexacaftor/tezacaftor/ivacaftor
HR: Heart rate
O_2_: Oxygen
*p*-value: Probability value
ppFEV_1_: Percent predicted forced expiratory volume in one second
rpm: Revolution per minute
RQ: Respiratory quotient
RR: Respiratory rate
SD: Standard deviation
V/Q: Ventilation/perfusion
VCO_2_: Carbon dioxide elimination
V_D_: Dead space
V_E_: Minute ventilation
V_T_: Tidal volume
V_E_/VCO_2_: Ventilatory equivalent for carbon dioxide
V_E_/VO_2_: Ventilatory equivalent for oxygen
VO_2_: Oxygen uptake
VO_2VT1_: Oxygen uptake on the first ventilatory threshold
VO_2VT2_: Oxygen uptake on the second ventilatory threshold
W: Watt
WR: Work rate
